# Surgically treated gastric melanoma of unknown primary: A case report from a 10‐year survivor

**DOI:** 10.1111/pin.12991

**Published:** 2020-08-16

**Authors:** Toshiyuki Takahashi, Shintaro Sugita, Hidetoshi Kagaya, Takayuki Morita

**Affiliations:** ^1^ Department of Pathology Hokkaido Gastroenterology Hospital Hokkaido Japan; ^2^ Department of Surgical Pathology, Sapporo Medical University School of Medicine Hokkaido Japan; ^3^ Department of Internal Medicine Hokkaido Gastroenterology Hospital Hokkaido Japan; ^4^ Department of Surgery Hokkaido Gastroenterology Hospital Hokkaido Japan

**Keywords:** CD117, gastric melanoma, long‐term survival, surgical treatment

## Abstract

We describe an extremely rare case of simultaneous double melanoma of the stomach with no other obvious primary source. The patient has survived for more than 12 years post‐complete gastrectomy. The patient was a woman in her seventies who was referred for anemia by another clinic. Esophagogastroscopy revealed an ulcerated gastric tumor that was diagnosed as a gastrointestinal stromal tumor (GIST) by endoscopic biopsy. She was admitted to our hospital for further examination and treatment. Gastroscopy at our institution revealed two submucosal tumors in the gastric wall. Since no metastatic lesions were detected after systemic exploration, multiple GIST of the stomach was diagnosed, and a total gastrectomy was performed. Malignant melanoma was diagnosed in both lesions by a histopathological study of the resected stomach. The patient's postoperative progress was good, and thorough examination of the skin did not result in the discovery of any systemic neoplastic lesions which could be regarded as the source for the primary tumor. No anticancer treatments were used. The patient has survived, with no recurrence for over ten years postsurgery. Strong evidence is presented in this case for the diagnosis and treatment of gastric malignant melanoma.

**Abbreviations**
GIST: gastrointestinal stromal tumor
H&E: hematoxylin and eosin

## INTRODUCTION

Malignant melanoma arising in the gastrointestinal canal is relatively rare. This disease occurs most frequently at the anorectal site, followed by cases in the esophagus.^1^ Primary malignant melanoma of the stomach is truly uncommon and approximately 20 cases have been described in the literature to date. In addition, the occurrence of primary malignant melanoma of the stomach is controversial, since it is thought that melanocytes do not originate in the gastric wall. Because many gastrointestinal malignant melanoma cases are accompanied by metastatic lesions of other organs at diagnosis, surgical treatment is not accepted for most patients. In surgically treated patients, there may not be a good prognosis for early recurrence or metastases to the other sites.^1^ Herein, we report that a patient with gastric malignant melanoma was surgically treated and survived for over 10 years posttreatment. We discuss the gastric malignant melanoma status of our case, as primary versus metastatic, and pitfalls of the pathological diagnosis of malignant melanoma on the digestive tract.

## CLINICAL SUMMARY

**Figure 1:**
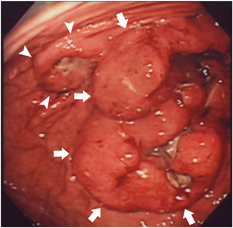
Endoscopic findings. Gastroscopy revealed two ulcerated masses on the body of the stomach. The larger mass is over 100 mm in diameter (arrows) and the smaller mass is approximately 30 mm (arrow heads).

A 75‐year‐old woman was referred to our hospital for a complete examination and surgery to treat a gastric tumor. The tumor was discovered via gastroscopy while investing causes of anemia diagnosed at the referring clinic. She had been diagnosed with a GIST for which histological results confirmed a CD117 (c‐kit) positive tumor via immunohistochemical staining. Although she had been medicated for hypertension for approximately 10 years, she had no other medical history, including any cutaneous diseases. A routine hematological study performed at admission showed hypochromic microcytic anemia. Both liver and kidney functions were within normal ranges. Tumor markers including carcinoembryonic antigen and carbohydrate antigen 19‐9 were also unremarkable. A gastroscopy was performed at our institution and revealed a large ulcerated tumor, over 100 mm in diameter, located on the greater curvature of the upper body of the stomach (Fig. 1). Simultaneously, another tumor with a central ulceration, 30 mm in diameter, was discovered on the anterior wall near the mass described above (Fig. 1). Because GIST was pathologically diagnosed at the previous clinic, a biopsy was not performed due to the risk for bleeding. Since whole body computed tomography and positron emission tomography showed no other lesion aside from the stomach, a total gastrectomy was performed.

## PATHOLOGICAL FINDINGS

**Figure 2:**
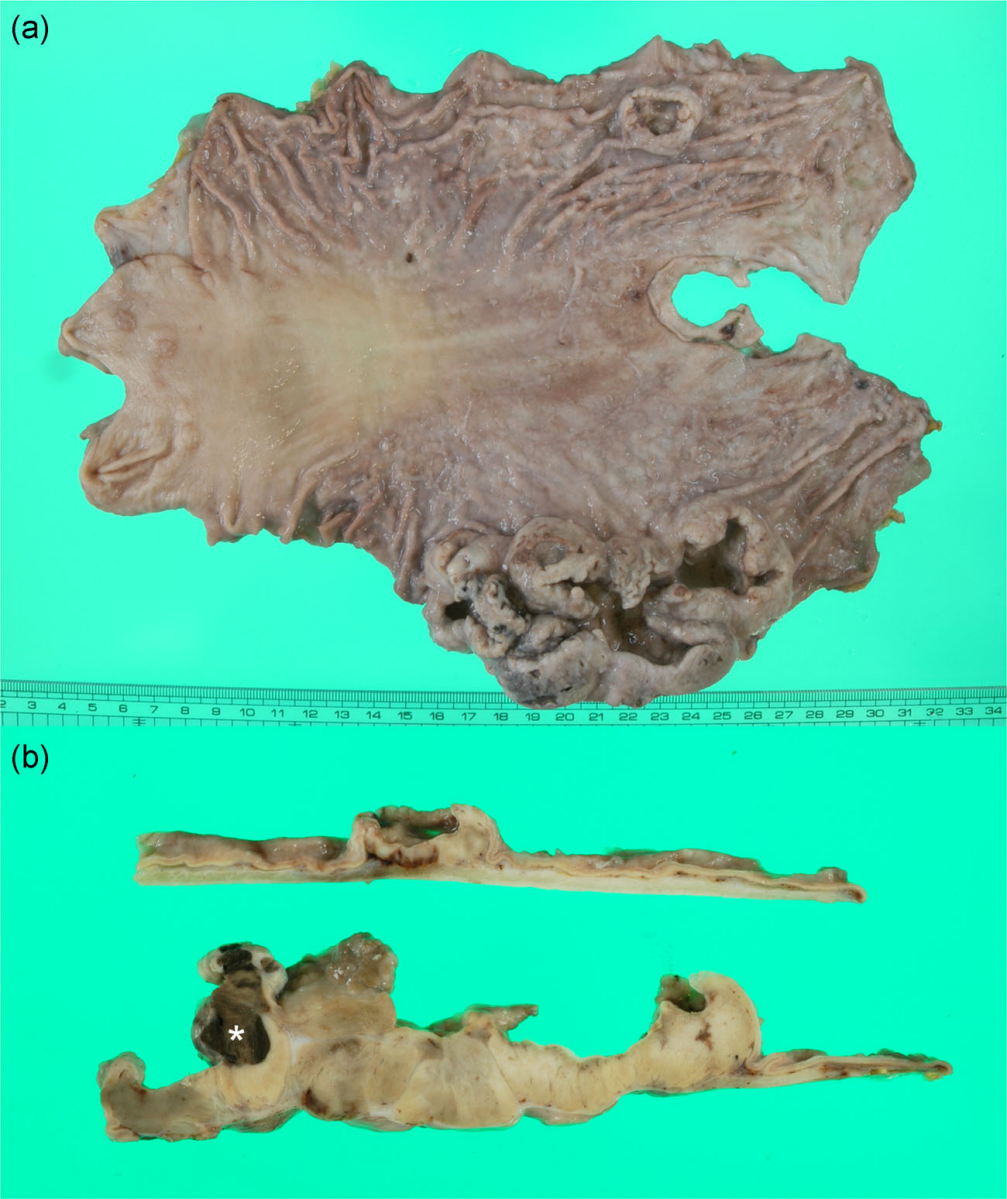
Gross and cut surface findings of the resected stomach. (**a**) Two lesions are noted on the mucosa, as on the endoscopic findings. A large mass, 115 mm in diameter, was seen on the greater curvature of the upper to middle body of the stomach. Another one, 26 mm in diameter, was on the anterior site of the above tumor. (**b**) Both tumors were yellowish gray in color with a focal dark brown area (asterisk) on the cut surface. Necrosis was not observed.

Two lesions were noted on the mucosa of the resected stomach by gross examination, as on the endoscopic findings (Fig. 2a). A large ulcerated mass, 115 mm in maximal diameter, on the greater curvature of the upper to middle body, was observed. In addition, an ulcerated delimitative tumor, 26 mm in diameter, was detected on the anterior site of the mass described above (Fig. 2a). These tumors were yellowish gray in color with a focal dark brown region on the cut surface (Fig. 2b). A necrotic area was not observed.

**Figure 3:**
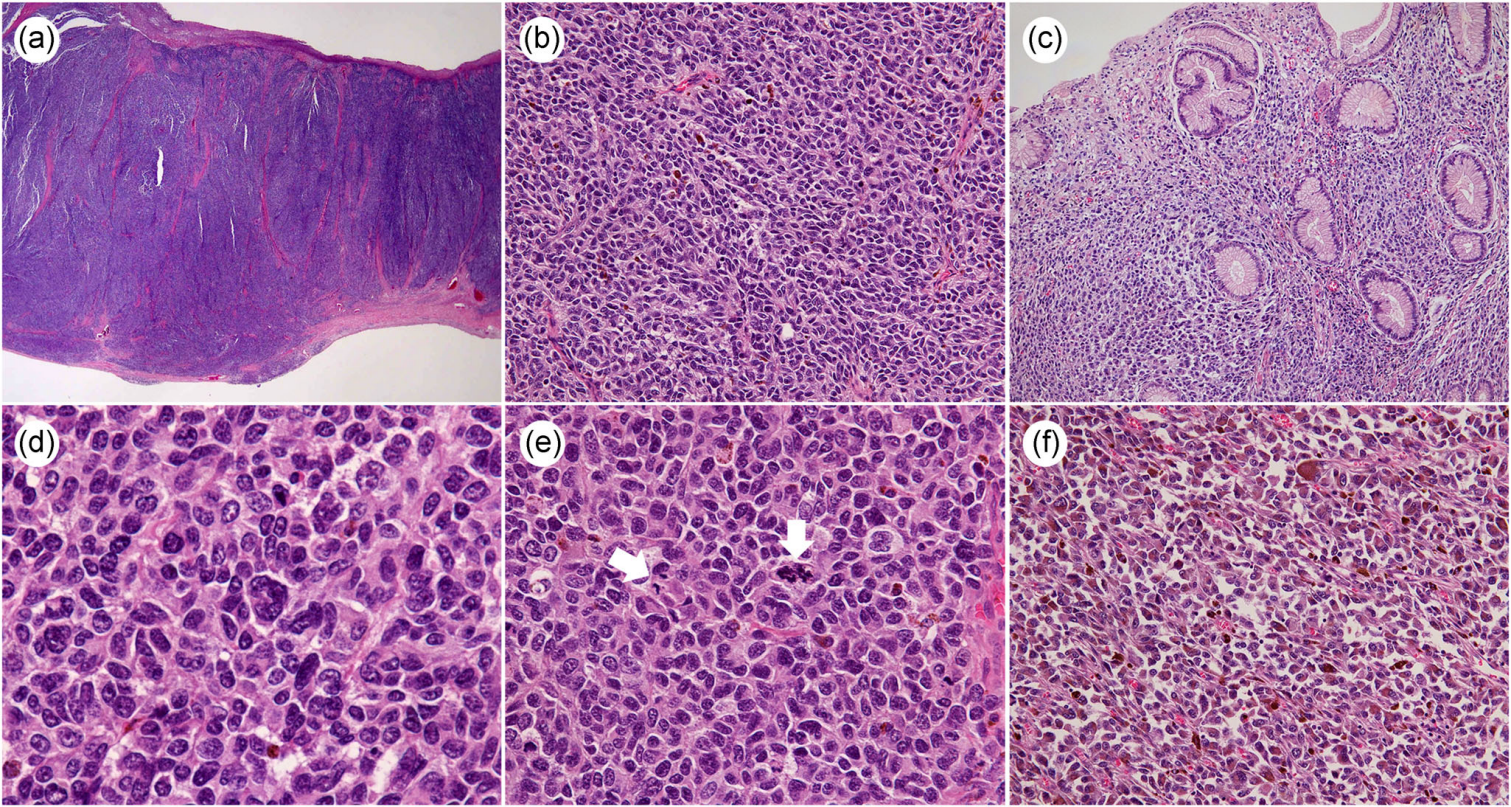
Microscopic findings of the tumors. (**a**) Neoplastic cells involved the muscularis propria to the submucosal layer under low‐power magnification (H&E). (**b**) Tumor cells grew in a sheet like fashion accompanied by scarce stroma (H&E, original magnification ×200). (**c**) The cells also invaded into the mucosa near the ulcer bed (H&E, original magnification ×100). (**d**) Individual cells showed irregular, enlarged nuclei containing rough chromatin and polygonal or spindle cytoplasm with high pleomorphism. Multinucleated cells could be observed at the center of the photograph (H&E, original magnification ×600). (**e**) Mitotic figures were often observed under high‐power magnification (H&E, original magnification ×400). (**f**) Many cells contained tiny tan granules in the cytoplasm, especially in the dark brown region (H&E, original magnification ×400).

**Figure 4:**
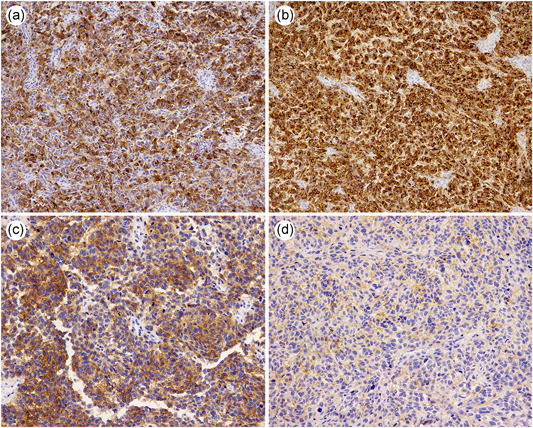
Immunohistochemical findings of the tumors. (**a** and **b**). Tumor cells were diffusely positive for HMB‐45 (**a**), and Melan A (**b**). (**c** and **d**) Cytoplasmic staining with cell‐membrane accentuation using c‐kit was observed in the large tumor (**c**), while faint membranous staining was seen in the small tumor (**d**).

Both tumors exhibited similar features upon histological examination. H&E stained slides under low‐power magnification showed that the neoplastic cells chiefly involved muscularis propria and the submucosal layer (Fig. 3a). Tumor cells mainly grew in a sheet, accompanied by scarce stroma, scattering a few cells including brown pigments in the cytoplasm (Fig. 3b). Although they were arranged in alveolar or lined up in a cord‐like pattern, no glandular formations were observed. The histological features we observed were similar to the endoscopic biopsy specimen taken at the previous clinic. The cells also invaded the mucosa neighboring the ulcer bed (Fig. 3c). Upon histological examination, individual cells could be classified as having high atypia. They had irregular, enlarged nuclei containing rough chromatin and polygonal or spindles in the cytoplasm. Some multinucleated or bizarre nucleated cells were intermixed within these cells (Fig. 3d). Mitotic figures were often observed under high‐power magnification (Fig. 3e). Many cells contained tiny tan granules in the cytoplasm, especially in the dark brown region of the anal site of the larger tumor (Fig. 3f). These histological findings are indicative of malignant melanoma. Tumor cells were diffusely positive for vimentin, human melanin black (HMB)‐45 (Fig. 4a), and melanocyte‐differentiation‐antigens MART‐1 (Melan A) (Fig. 4b) via immunohistochemistry obtained from both lesions. Although neoplastic cells from both tumors were immunohistochemically positive for c‐kit, the immunohistochemical reaction of the large tumor was stronger than the small one. Cytoplasmic staining with cell‐membrane accentuation was observed in the large lesion (Fig. 4c), while faint membranous staining was seen in the small tumor (Fig. 4d). S‐100 protein showed focal, weak staining in both tumors. Cytokeratin, carcinoembryonic antigen, discovered on GIST (DOG‐1), desmin, CD34 and CD45 were all negative.

We examined specific gene rearrangements including Ewing sarcoma breakpoint region 1 (*EWSR1*), EWSR‐1 activating factor 1 (*ATF1*), and cAMP response element‐binding protein (*CREB1*) of the tumors to eliminate a possibility of clear cell sarcoma (CCS) and clear cell sarcoma‐like gastrointestinal tumor (CCSLGT). We used fluorescence *in situ* hybridization (FISH) to detect these rearrangements. FISH was performed as previously described.^2^ To detect the presence of *EWSR1, ATF1* and *CREB1*, we counted 100 nuclei in tumor cells and that showed a pair of fused and split signals, and calculated the percentage of split signals. We considered the specimen to be split positive if split signals were observed in more than 10% of tumor cells. Subsequently, split signals of *EWSR1, ATF1* and *CREB1* in tumor cells of the large lesion were 5%, 2% and 0%, respectively. They were below the cut off (10%). Concerning *EWSR1*, 95 cells showed one, two, three or four fused signals without any split signals. Four cells showed one fused and one split signal. One cell showed three fused and one split signal. Therefore, five cells were considered to be split ‘positive’. Concerning *ATF1*, 98 cells showed one, two or three fused signals without any split signals. Two cells showed one fused and one split signal. Therefore, two cells were considered to be split ‘positive’. Thus, the rearrangements were not detected. Unfortunately, fluorescent signals were not observed in tumor cells of the smaller lesion, since the paraffin embedded specimen deteriorated. Finally, a pathological diagnosis of gastric melanoma was identified for both lesions.

The patient's postoperative care was uneventful, and the patient was discharged on postoperative day 29. After discharge, detailed systemic examination by a dermatologist or ophthalmologist failed to detect any suspected melanoma lesions on the skin or eye. The patient's follow‐up care was carefully monitored using whole body computed tomography scans yearly, up to 5 years postsurgery and by visual inspection and interview after the sixth year. As of 146 months postsurgery, she remains alive without significant recurrence.

## DISCUSSION

The National Cancer Database was analyzed to examine melanoma cases diagnosed between 1985 and 1994, and they reported that the vast majority of melanomas originate in the skin (91.2%), with the next highest percentage being ocular (5.3%). The mucous membrane was only responsible for 1.3% of all melanoma sites.^3^ Among mucosal melanomas, the incidence of head and neck was 55.4% and that of the anal/rectal region was 23.8%, respectively.^3^ Cheung *et al*.^1^ explained that anorectal lesions accounted for over 50% of the 659 cases of gastrointestinal melanoma studied, followed by oral‐nasopharynx in over 30% of these cases. Esophagus, stomach, small intestine and colon lesions were responsible for 5.9%, 2.7%, 2.3% and 0.9% of the cases, respectively.^1^ Thus, gastric melanoma, whether primary or metastatic, is an extremely rare entity.

Both CCS and CCSLGT have some similar pathologic characteristics. They are often positive for melanoma markers, such as HMB‐45, Melan A, as well as S‐100 protein.^4^, ^5^ These tumors, therefore, are sometimes difficult to differentiate from malignant melanoma. CCS and CCSLGT are known to express chimeric fusions of *EWSR1, ATF1* and *CREB1* in various degrees.^2^, ^4^, ^5^ Thus, examination of the genetic rearrangement of melanin producing tumors is useful for differential diagnosis of CCS/CCSLGT and malignant melanoma. Since these rearrangements were not detected in our case, the lesion was consequently diagnosed as malignant melanoma.

Since melanocytes or melanosis, a benign counterpart of malignant melanoma, are not usually found in the gastric mucosa, it is controversial whether primary melanoma of the stomach truly exists. Furthermore, malignant melanoma of the skin often metastasizes to the gastrointestinal canal. An autopsy series from Roswell Park suggests that the gastrointestinal system is second only to the lung in frequency of metastatic disease, with an incidence of 43.5%.^6^ Similarly, in a large autopsy review, the incidence of gastrointestinal metastases was as follows: liver 68%, small bowel 58%, colon 22%, stomach 20%, duodenum 12%, rectum 5%, esophagus 4% and anus 1%.^7^ Thus, melanomas of the gastrointestinal canal should be examined to distinguish primary lesions from metastases. The criteria for diagnosing primary gastric melanomas includes: (i) a single melanoma lesion located in the stomach with proven pathology; (ii) no concurrent lesions in other sites of the body; (iii) no history of melanoma; (iv) disease‐free survival of at least 12 months after curative surgery.^8^, ^9^ Although our patient satisfies (ii), (iii) and (iv) of the above criteria, this case is different from other published reports, as this patient presents with primary gastric melanoma that has resulted in a simultaneous, double tumor in the gastric wall. We formulate three hypotheses concerning whether these two melanomas are primary or metastatic. The first is that each tumor is primary. The second is that both lesions are metastatic, resulting from another site. An additional hypothesis is that the larger tumor is primary and the smaller one is its intramural metastasis. Concerning the first hypothesis, it is difficult to believe the likelihood for two such rare neoplasms to occur simultaneously. Approximately 2% of the over 80,000 cases of cutaneous and noncutaneous melanomas were reported to have an unknown primary site when deposited in the National Cancer Database.^3^ Some authors have also described gastric melanoma cases of unknown primary origin.^10^, ^11^ In these reports, the authors indicate that the primary tumor might not be noted in the history, because skin melanomas can sometimes spontaneously regress. It is likely that both lesions are metastases from the other primary site, even if postoperative exploration did not disclose melanoma lesions on the skin systemically. The remaining hypothesis is that the large lesion is the primary gastric melanoma and the small lesion is its intramural metastasis. However, we really cannot trust this hypothesis, unless the existence of melanocytes in the gastric mucosa is proved. Consequently, it may be difficult to identify the accurate primary site in this case. Cases similar to our patient where there are more than two melanomas in the stomach but no lesions present in other organs have never been reported. This point makes our case unique.

Here, we will summarize reasons for failing to identify the melanoma preoperatively. First, both physicians who performed the endoscopy and the pathologist who made the diagnosis were unaware of the major cause. C‐kit, which is one of immunohistochemical markers for GIST was positive. In this case, that might confuse the pathological diagnosis from the first biopsy. Potti *et al*. showed 22.8% of 202 cutaneous melanoma cases were immunohistochemically positive for c‐kit.^12^ They described that c‐kit was overexpressed in 53.7% of cases for superficially spreading melanoma, indicating that c‐kit is overexpressed primarily in early stages of disease, although there was no statistically significant difference in the survival between the c‐kit‐positive and c‐kit‐negative groups.^12^ Since it has been described that mucosal melanomas appear to have high incidence of activating mutations and/or amplifications in the KIT oncogene rather than cutaneous melanoma,^13^ pathologists should be careful for c‐kit positivity in diagnosis of mesenchymal tumor of the gastrointestinal tract. We should also recognize that multiple occurrence of GIST is unusual, even if we suspected GIST. Furthermore, we had to perceive the immunohistochemical finding which was positive for S‐100 suggested the possibility of melanoma.

If we could correctly diagnose this case as melanoma before surgery, we might select some strategies for treatment. Radiation is useful for most of these cases in the palliative setting to control symptomatic local disease.^13^, ^14^ However, most studies have failed to demonstrate an improvement in overall survival with adjuvant radiotherapy, although these findings are complicated by the fact that there is tendency for radiotherapy to be favored in more advanced cases.^13^ Effects of systemic therapy are also limited. Even still, with limited data available, no systemic therapy has been shown to significantly improve outcomes.^13^, ^15^ Several trials examining effect of combination chemotherapy for the disease are ongoing.^13^ In addition, systemic therapy options including targeted therapies, and immunotherapies in the literature.^13^, ^15^, ^16^ We will be able to select these agents for future therapeutics. Surgery remains the primary therapeutic intervention for mucosal melanoma by recent some reviews^13^, ^16^ regardless of primary or metastatic lesion. Wong *et al*.^17^ reported a 5‐year survival rate of 20% in 144 patients who underwent surgical resection of non‐regional melanoma metastases. Cheung *et al*.^1^ performed surgical extraction for 82.1% of 652 patients diagnosed with primary gastrointestinal melanoma and a median overall survival of 17 months. Their overall survival was better than nonoperative cases for 8 months. Their study recommended surgical therapy for patients without metastatic disease, since local disease exhibits statistically significant superior survival (median 30 months) when compared to patients with metastases to regional lymph‐nodes or distant organs (8 months).^1^ Akaraviputh *et al*.^18^ tried surgical intervention for 13 cases with gastrointestinal melanoma, whether they were primary or metastatic. This study reported that the most effective therapy is for lesions to be surgically removed when complete resection is possible, because the mean survival of the curative resected group is 29.7 months, longer than that of the palliative surgery group, 4.8 months. At the moment, we emphasize that surgical treatment should be indicated for patients with gastrointestinal melanoma with no other metastases, if the patient's systemic condition can endure the operation.

## DISCLOSURE STATEMENT

None declared.

## AUTHOR CONTRIBUTIONS

TT contributed drafting the manuscript, making pathologic photos. SS contributed FISH to detect gene rearrangement of tumor cells. HK contributed making endoscopic photograph and patient's medical data. TM advised TT the surgical technics and information.
